# Sleep Disordered Breathing and Its Predictors in Pediatric Muscular Dystrophies

**DOI:** 10.3390/jcm14227925

**Published:** 2025-11-08

**Authors:** Mahmoud Abu Zahra, Raanan Arens, Muhammed Amir Essibayi, Neha Patel

**Affiliations:** 1 Division of Pediatric Respiratory and Sleep Medicine, The Children’s Hospital at Montefiore, Albert Einstein College of Medicine, Bronx, NY 10467, USA; raanan.arens@einsteinmed.edu; 2 Department of Neurological Surgery, Montefiore Medical Center, Albert Einstein College of Medicine, Bronx, NY 10467, USA; muhammedamir.essibayi@einsteinmed.edu; 3 Department of Pediatric Respiratory and Sleep Medicine, Newark Beth Israel, New Jersey Medical School, Newark, NJ 07112, USA; neha.patel3@rwjbh.org

**Keywords:** sleep disordered breathing, obstructive sleep apnea, hypoventilation, muscular dystrophy, BiPAP, polysomnography, pulmonary function test

## Abstract

**Background/Objectives:** To evaluate the prevalence, age at diagnosis, non-invasive ventilation pressures used in management, and clinical predictors for sleep disordered breathing (SDB) in pediatric patients with muscular dystrophies (MDs). **Methods:** A retrospective analysis of 195 polysomnography (PSG) studies conducted over 20 years for 98 children with different MDs was performed. Diagnosis of SDB was established if a child met the diagnostic criteria for one or more of the following conditions: obstructive sleep apnea (OSA), central apnea, nocturnal hypoxemia, or nocturnal hypoventilation. Outcomes were assessed and compared between MDs. Positive and negative predictive values (PPV, NPV), sensitivity, and specificity for detecting SDB were calculated for certain clinical parameters. **Results:** SDB was diagnosed in 73.6% of children with MDs, including OSA in 67%, followed by nocturnal hypoxemia (15.3%), nocturnal hypoventilation (7.7%), and central apnea (6.6%). The age at diagnosis and BiPAP pressures used varied between MDs. Patients with Congenital MD had the lowest mean age and required higher pressures (*p* < 0.05). PPV was high for maximum inspiratory or expiratory pressures (MIP, MEP) < 40% or <60%, forced vital capacity < 50% or <80%, total lung capacity < 60%, left ventricular ejection fraction < 50%, non-ambulation, and body mass index ≥ 95% for the presence of SDB. However, NPV, sensitivity, and specificity varied. **Conclusions:** SDB is common in pediatric patients with MDs, with OSA being the most prevalent disorder. The age at diagnosis and required BiPAP pressures for management differ among MD groups. Certain clinical measures may help identify some patients with the disease given the high PPV.

## 1. Introduction

Muscular dystrophies (MDs) comprise a group of genetic disorders leading to generalized progressive muscular weakness and atrophy [1,2]. Different muscles are frequently involved in these diseases including the diaphragm, cardiac, neck, upper airway, and facial muscles [1,2]. As such, affected children may develop overtime respiratory insufficiency, cardiomyopathy, and sleep disordered breathing (SDB) [1,3,4].

Various types of MDs have been described including Congenital, Duchenne, Becker, Facioscapulohumeral, Limb-girdle, Myotonic, and Emery–Dreifuss [1,2,5]. Congenital MD is a group of genetic diseases that typically present with signs at birth or within the first few months of life [6]. The weakness is severe and progressive with most patients unable to achieve ambulation [1,6]. Duchenne MD is an X-linked disease with mutation leading to diminished dystrophin production in muscles causing progressive loss of muscle tissue and function, with symptoms appearing around the age of 2–3 years [7]. Becker MD belongs to the same group of dystrophinopathies as Duchenne, but its mutation results in a partial loss of function of dystrophin, with a slower progression and later onset of symptoms [1,8]. Facioscapulohumeral MD is an autosomal dominant disorder with a mutation-activating DUX4 gene leading to weakness in the face, shoulders, and upper arms [1,9]. Limb-girdle MD is a diverse group of diseases presenting with proximal muscle weakness around the hips and shoulders with varying clinical course and genetic mutations, inherited in either an autosomal recessive (most common) or dominant pattern [1,10]. Myotonic MD is an autosomal dominant disorder caused by abnormal DNA expansion in the DMPK gene (type 1) or CNBP gene (type 2), causing weakness, myotonia, and cataracts [11]. Emery–Dreifuss is a rare MD which presents around the age of 10 years with wasting and weakness involving the shoulders, upper arms, and calf muscles, and is most commonly inherited as an X-linked recessive disease [12].

Although SDB has been reported in children with MDs [3,4,13,14,15], the differences in prevalence, severity, onset of the disease, and how patients at risk could be identified, are not well addressed, particularly among the less common MDs. This study aimed to determine the prevalence, age at diagnosis, non-invasive ventilation pressures used in management, and clinical factors associated with SDB in children with different MD types. We hypothesized that SDB prevalence and onset vary among MD types and that specific clinical features can help identify those at higher risk. To test this hypothesis, we conducted a retrospective medical chart review over the past 20 years at a tertiary care center.

## 2. Materials and Methods

### 2.1. Subjects

We searched the data saved from in-laboratory PSG studies performed at The Children’s Hospital at Montefiore (New York, USA), between January 2004 and September 2023 for patients with MDs. A total of 195 studies for 98 patients aged ≤ 21 years with confirmed MD diagnosis were identified and included. Patients with MDs are routinely screened for SDB in our center, and the referrals for PSG were placed by pediatric pulmonologists, pediatric neurologists, or primary care providers. MD diagnosis was confirmed by reviewing medical charts from each child’s pediatric neurologist, who established the diagnosis based on thorough neurological assessment, genetic testing (performed in all patients), and muscle biopsy. The study was approved by the Institutional Review Board of Albert Einstein College of Medicine No. 2025-17127.

### 2.2. Data and PSG

Medical charts reviewed included inpatient and outpatient encounters, PSG reports, pulmonary function test (PFT) results, and echocardiography reports. PFT results were included in the analysis if performed within 6 months of the sleep study, and echocardiography measures were included if performed within 12 months of the sleep study.

In-laboratory PSG studies included at least six channel electroencephalograms, two electro-oculograms, submental and bilateral tibialis surface electromyograms, and an electrocardiogram. Additional recordings included airflow from nasal pressure and nasal/oral thermocouple, chest and abdominal movement via respiratory impedance plethysmography belts, end-tidal CO_2_ via a capnograph sampled through a nasal cannula, and blood oxygen saturation via a finger probe. Time-locked digital video was recorded with the PSG. The multichannel polysomnogram was recorded digitally and was initially scored by an American Academy of Sleep Medicine board-certified sleep technician, then reviewed and finalized by a board-certified sleep physician.

Diagnosis of SDB was made if a child had one or more of the following: obstructive sleep apnea (OSA), central apnea, nocturnal hypoxemia, or nocturnal hypoventilation in at least one of their PSG studies [16,17], based on the International Classification of Sleep Disorders Third Edition ICSD-3 criteria [18]. Obstructive sleep apnea was diagnosed if the patient had an obstructive apnea/hypopnea index (OAHI) of ≥1 for patients below the age of 18 years and if OAHI was ≥5 for patients 18 years and older [16,19]. OSA severity was classified based on the OAHI. For those younger than 18 years old, mild, moderate and severe were defined, respectively, by the following ranges: 1 ≤ OAHI < 5, 5 ≤ OAHI < 10 and OAHI ≥ 10; for those at or above 18 years old, the corresponding ranges were 5 ≤ OAHI < 15, 15 ≤ OAHI < 30, and OAHI ≥ 30 [16,19]. A diagnosis of central apnea was made if the patient had an central apnea index (CAI) of ≥5 [20]. Nocturnal hypoxemia was defined by oxygen saturation below 90% for more than 2% of total sleep time (TST) or for a continuous 5 min [16,21,22]. Nocturnal hypoventilation was defined by end tidal carbon dioxide (ETCO_2_) > 50 mmHg for more than 25% of TST [16]. For patients who underwent more than one PSG study, the age at diagnosis was determined based on their age at the time of the earliest PSG confirming SDB. Diagnostic measures for SDB were extracted from baseline PSG studies and the first part (baseline portion) of split night studies. Non-invasive respiratory support measures were extracted from the latter portion of split night studies (after initiating non-invasive support) and from titration studies. Data included from PSG studies: age, sex, height, weight, body mass index (BMI), CAI, OAHI, baseline oxygen saturation, minimum oxygen saturation, baseline ETCO_2_, maximum ETCO_2_, TST percentage with O_2_ saturation between 90–100%, TST percentage with O_2_ saturation between 80–90%, TST percentage with O_2_ saturation below 80%, TST percentage with ETCO_2_ between 0–50 mmHg, TST percentage with ETCO_2_ > 50 mmHg, PSG study type (baseline, split night, or titration), CPAP, BiPAP, ventilator pressures, oxygen flow, and fractional inspired oxygen (FiO_2_). From medical charts: race, MD type, lung disease, cardiac disease, echocardiography findings, left ventricular ejection fraction (LVEF), tonsillo-adenoidectomy (T&A), airway surgery, tracheostomy status, placement age, and indication, maximal inspiratory pressure MIP, maximal expiratory pressure (MEP), forced vital capacity (FVC), total lung capacity (TLC), and medications. Some PSG studies and medical charts did not document all of these variables; thus, our dataset has some sporadic missing values.

### 2.3. Statistical Analysis

We classified BMI categories based on centiles for patients < 18 years of age as follows: underweight < 5th centile, healthy weight 5th to 85th centile, overweight 85th to 95th centile, and obese at 95th centile or greater [23]. For patients ≥ 18 years old, categories were classified based on BMI values as follows: underweight < 18.5, healthy weight 18.5 to less than 25, overweight 25 to less than 30, and obese 30 or greater [24]. Continuous variables were summarized as mean ± standard deviation (SD) and analyzed using the t-test for pairwise comparisons and one-way Analysis of Variance (ANOVA) for multiple group comparisons [25]. Categorical variables were presented as frequencies and percentages, with statistical comparisons performed using Fisher’s exact test [26]. Sensitivity, specificity, positive predictive value, and negative predictive value were calculated using standard formulas [27]. A *p*-value < 0.05 was considered statistically significant. All analyses were conducted using Jamovi software (Version 2.5.7; The Jamovi Project, Sydney, Australia).

## 3. Results

### 3.1. Patients’ Characteristics (Table 1)

The study included 98 patients with 7 different MDs. The most prevalent MD type was Duchenne (43.9%, n = 43). The mean age of the group was 11.7 years, the youngest patient was 1 month old, and the oldest was 21 years old. Males accounted for the majority of the sample (75.5%) which is explained by the X-linked mode of inheritance in certain MDs. The mean BMI was 21.2 kg/m^2^ and the average BMI percentile was 60%. When classified by BMI category, most subjects were in the healthy weight class (38.8%). No significant differences were found between the different MD types in terms of BMI categories distribution.

**Table 1:** Patients’ characteristics.

**Muscular Dystrophy**	**n (%)**
Duchenne	43 (43.9%)
Myotonic	18 (18.4%)
Congenital	15 (15.3%)
Limb-girdle	11 (11.2%)
Becker	7 (7.1%)
Facioscapulohumeral	3 (3%)
Emery–Dreifuss	1 (1%)
**Age**	**Years**
Mean (SD)	11.7 (5.4)
Range	0.1–21.0
**Sex**	**n (%)**
Female	24 (24.5%)
Male	74 (75.5%)
**Race**	**n (%)**
Black	21 (21.4%)
White	14 (14.3%)
Asian	4 (4.1%)
Native Hawaiian or Pacific Islander	2 (2%)
Other/Unspecified/Declined	57 (58.2%)
American Indian or Alaska Native	0 (0%)
**BMI**	**Kg/m^2^**
Mean (SD)	21.2 (7.9)
Range	7.2–58.3
**BMI percentile**	**%**
Mean (SD)	60% (40)
Range	0.0–100%
**BMI categories**	**n (%)**
Underweight	17 (16.5%)
Healthy weight	33 (38.8%)
Overweight	15 (17.6%)
Obese	23 (27.1%)

SD standard deviation, n total number of patients, % percentage within the group, BMI body mass index. Bold is used for headings within the table.

### 3.2. Clinical Data (Table 2)

Lung disease was present in 26.3% of the patients, with asthma being the most common (22.5%). Cardiac conditions were reported in 29.7%, with dilated cardiomyopathy being the most common. The mean LVEF was 57%. Ambulatory status was documented in 71 patients, with 63.5% being ambulatory at the time of the sleep study. Only three patients had airway surgery prior to performing the sleep study; one patient, a Congenital MD patient, had a tracheostomy placed for chronic respiratory failure, and two patients had T&A. Medications were documented for 73 patients: 59% took no medications for MD, 35.6% used steroids only (23 Duchenne and 3 Becker patients), and combination therapy with steroids and Golodirsen, Eteplirsen, or Casimersen was reported in 5.4% (4 patients with Duchenne). Pulmonary function testing for the patients showed a mean value of 81% for FVC% and 86% for TLC%. Depressed respiratory muscle strength was noted, the MIP% mean was 45%, and the MEP% mean was 26%.

**Table 2:** Patients’ clinical information.

**Lung Disease**	**n (%)**
Recurrent aspiration	2 (2.5%)
Asthma	18 (22.5%)
Interstitial lung disease	1 (1.3%)
**Cardiac Disease**	**n (%)**
1st degree heart block	1 (1.4%)
PDA	1 (1.4%)
PFO	1 (1.4%)
VSD	1 (1.4%)
Dilated aortic root	1 (1.4%)
DCM	14 (19%)
DCM underwent heart transplant	1 (1.4%)
Pulmonary hypertension	1 (1.4%)
**LVEF**	**%**
Mean (SD)	57% (9.4)
Range	20.9–70.6%
**Ambulatory Status**	**n (%)**
Yes	47 (63.5%)
No	27 (36.5%)
**Medications**	**n (%)**
Steroids only	26 (35.6%)
Steroids + Eteplirsen	2 (2.7%)
Steroids + Casimersen	1 (1.4%)
Steroids + Golodirsen	1 (1.4%)
**FVC**	%*
Mean (SD)	81% (25)
Range	12–123%
**TLC**	%*
Mean (SD)	86% (22)
Range	53–123%
**MIP**	%*
Mean (SD)	45% (20)
Range	18–113%
**MEP**	%*
Mean (SD)	26% (13.7)
Range	3–75%

MIP and MEP analyzed in 47 subjects, FVC in 51, and TLC in 37. n total number of patients, % percentage within the group, DCM dilated cardiomyopathy, LVEF left ventricular ejection fraction, FVC forced vital capacity, %* percent predicted, TLC total lung capacity, MIP maximum inspiratory pressure, MEP maximum expiratory pressure. Bold is used for headings within the table.

### 3.3. Polysomnography Studies (Table 3)

A total of 195 PSG studies were included in the analysis for the ninety-eight patients with MDs. Forty-four patients underwent more than one study, including two patients who each had 7 studies. PSG studies included 118 baseline, 24 split nights, 44 BiPAP titration, 6 CPAP titration, one infant who had oxygen titration, and one tracheostomy-dependent patient with Congenital MD who had two PSG studies while on a ventilator. Split nights included 15 BiPAP titrations, 5 oxygen titrations, and 4 CPAP titrations performed in the latter part of the study.

**Table 3:** Polysomnography studies distribution by muscular dystrophy type.

Muscular Dystrophy Type	Patients	Sleep Studies	Baseline	Split Night	BiPAP Titration	CPAP Titration	Oxygen Titration	Ventilator
Duchenne	43	79	51	8	19	1	-	-
Myotonic	18	51	32	5	8	5	1	-
Congenital	15	30	11	5	12	-	-	2
Limb-girdle	11	17	12	3	2	-	-	-
Becker	7	14	8	3	3	-	-	-
Facioscapulohumeral	3	3	3	-	-	-	-	-
Emery–Dreifuss	1	1	1	-	-	-	-	-

### 3.4. Sleep Disordered Breathing (Table 4)

Sleep disordered breathing (SDB) was diagnosed in at least one study for 67 patients (73.6%) with MDs, and the mean age at diagnosis was 11.6 years. OSA was the most common, being diagnosed in 61 patients (67%). A total of 14 patients (15.3%) had nocturnal hypoxemia, 7 (7.7%) had nocturnal hypoventilation, and 6 (6.6%) had central apnea. Among those with OSA, 61% had mild OSA, 21% had moderate, and 18% had severe. The distribution of these variables by MD type is shown in Table 4.

**Table 4:** Sleep disorder breathing and age at diagnosis in muscular dystrophies.

	SDB (n = 67, 73.6%)		OSA (n = 61, 66%)		Central Apnea (n = 6, 6.6%)		Nocturnal Hypoxemia (n = 14, 15.3%)		Nocturnal Hypoventilation (n = 7, 7.7%)	
**Mean age (SD)**	11.6 (5.1)		12.0 (4.8)		12.7 (7.2)		12.5 (6.2)		11.1 (4.9)	
**Age range**	0.1–21.0		0.3–21.0		0.3–21.0		0.1–20.8		4.5–19.2	
**MD type**	**n (%)**	**Age**	**n (%)**	**Age**	**n (%)**	**Age**	**n (%)**	**Age**	**n (%)**	**Age**
Congenital	10 (77)	7.3	8 (62)	8.1	1 (8)	0.3	4 (27)	8.9	0 (0)	NA
Emery–Dreifuss	1 (100)	4.4 *	1 * (100)	4.4	0 (0)	NA	0 (0)	NA	0 (0)	NA
Becker	4 (57)	12.8	4 * (57)	12.8	0 (0)	NA	2 (29)	15.5	0 (0)	NA
Duchenne	31 (78)	12.9	30 (75)	12.8	1 (3)	14.3	5 (12)	17.1	4 (11)	11.8
Facioscapulohumeral	2 (67)	9.5 *	2 * (67)	9.5	0 (0)	NA	0 (0)	NA	0 (0)	NA
Limb-girdle	6 (55)	11.3	6 (55)	11.3	0 (0)	NA	0 (0)	NA	1 (11)	11.3
Myotonic	13 (77)	12.4	10 (59)	13.9	4 (24)	15.4	3 (17)	7.6	2 (13)	9.6
*p* value		0.032		0.032						

Age in this table refers to the age at diagnosis. * values not included in the analysis for age at diagnosis. SDB sleep disordered breathing, n total number of patients, % percentage within the group, OSA obstructive sleep apnea, SD standard deviation, MD muscular dystrophy, NA not applicable. Bold is used for headings within the table.

Multiple pairwise and group comparisons were performed to evaluate for significant differences in age at diagnosis between the various MDs. When comparing Congenital, Myotonic, Limb-girdle, Duchenne, and Becker using ANOVA, the differences were statistically significant (*p*-value 0.032), and patients with Congenital had the lowest mean age at diagnosis of SDB, namely 7.3 years. Significant differences (*p*-value 0.032) were also detected in age at diagnosis when comparing OSA between Myotonic, Limb-girdle, Duchenne, and Congenital, which also had the lowest mean age of 8.1 years. Pairwise comparisons revealed significant differences in age at diagnosis for SDB and OSA when comparing Congenital with Duchenne or with Myotonic patients.

### 3.5. Non-Invasive Ventilation (Table 5)

BiPAP was used in 59 studies, including 44 titrations and 15 split night studies. The mean positive end expiratory pressure (PEEP) was 6.5 cmH_2_O, and the mean peak inspiratory pressure (PIP) was 11.9 cmH_2_O. When comparing Congenital, Becker, Duchenne, Limb-girdle, and Myotonic groups, significant differences were present, and patients with Congenital required higher BiPAP pressures. CPAP, oxygen via nasal cannula, and ventilators were used in a limited number of patients, which did not allow for meaningful comparison.

**Table 5:** BiPAP pressures used to manage sleep disordered breathing in muscular dystrophies compared.

BiPAP Pressure	Congenital (n = 14)	Becker (n = 6)	Duchenne (n = 26)	Limb-Girdle (n = 4)	Myotonic (n = 10)	*p* Value
PEEP mean (SD)	8.1 (3.2)	5.5 (0.8)	6.1 (1.4)	5.5 (0.6)	6.6 (1.4)	0.016
PEEP range	4.0–12.0	4.0–6.0	4.0–9.0	5.0–6.0	5.0–9.0	
PIP mean (SD)	14.3 (4.7)	9.7 (0.8)	11.5 (2.5)	10.0 (0.0)	11.5 (2.1)	0.01
PIP range	8.0–23.0	8.0–10.0	8.0–17.0	10.0–10.0	9.0–15.0	

n total number of patients, PEEP positive end expiratory pressure, SD standard deviation, PIP positive inspiratory pressure.

### 3.6. Predictors of Sleep Disordered Breathing (Table 6)

We calculated the sensitivity, specificity, PPV, and NPV of certain clinical parameters to predict the presence of SDB. The parameters included LVEF < 50%, if the patient is non-ambulatory (did not achieve ambulation or lost the ability to ambulate), BMI% ≥ 95%, MIP < 40%, MIP < 60%, MEP < 40%, MEP < 60%, FVC < 50%, FVC < 80%, TLC < 60%, and TLC < 80%. All parameters had a PPV higher than 80% except for TLC < 80%. The highest PPV, 91.3%, was for MIP < 40%. NPV was low for all the predictors. Sensitivity and specificity varied; MEP < 60% had the highest sensitivity, 97.4%, but the specificity was 0% due to lack of true negative values. TLC < 60% and FVC < 50% had the highest specificity, but their sensitivity was limited.

**Table 6:** Clinical predictors for sleep disordered breathing.

Clinical Measure	Sensitivity (%)	Specificity (%)	PPV (%)	NPV (%)
LVEF < 50%	17.6	85.7	83.3	20.5
Non-ambulatory	40.4	73.1	84.4	25.3
BMI% ≥ 95	39.2	76.9	86.4	25.3
MIP < 40%	53.8	75	91.3	25
MIP < 60%	82.1	37.5	86.5	30
MEP < 40%	87.2	25	85	28.6
MEP < 60%	97.4	0	82.6	0
FVC < 50%	11.6	87.5	83.3	15.6
FVC < 80%	44.2	62.5	86.4	17.2
TLC < 60%	20.7	87.5	85.7	23.3
TLC < 80%	58.6	37.5	77.3	20

Non-ambulatory includes patients who didn’t achieve ambulation or lost the ability to ambulate. Percent predicted used for pulmonary function test results including MIP, MEP, FVC, and TLC. Specificity and NPV for MEP < 60% is 0 due to absence of true negative values. PPV positive predictive value, NPV negative predictive value, LVEF left ventricular ejection fraction, BMI% body mass index percentile, MIP maximum inspiratory pressure, MEP maximum expiratory pressure, FVC forced vital capacity, TLC total lung capacity.

## 4. Discussion

Our study revealed a high prevalence of SDB among children with various MD types who had polysomnography evaluation, and OSA was the most common, which is consistent with prior reports [3,4,13,14,28]. Nocturnal hypoventilation has been described in the literature as a classic complication of MDs [14,17,29], but in our study it was only diagnosed in 7.7% of the patients, and we believe this might have been affected by the relatively young mean age of the group, 11.7 years, as nocturnal hypoventilation needs more time to develop in these patients, typically presenting at an older age with advanced disease [17,30]. Age at diagnosis was different among the MD groups, which we believe correlates with the severity of the original disease and its rate of progression [2,14,17]. Patients with Congenital MD had the lowest mean age at diagnosis, 7.3 years, a finding justified by the early onset of symptoms and significant respiratory involvement early in the course of this illness [6]. In addition, the mean age at diagnosis was less than 13 years for all the types, which would signify the need for evaluating patients with MDs for SDB before adolescence.

The use of non-invasive ventilation (NIV), particularly BiPAP, has become a standard of care for managing SDB in MDs [3,4,29]. BiPAP has been used extensively for management of nocturnal hypoventilation in MD with studies reporting its benefits in prolonging survival and quality of life [4,17,31]. Among our group of patients, BiPAP was effective in improving oxygen saturation and in eliminating respiratory events during their sleep assessment. The pressures used varied, which seems to also correlate with the severity of their original MD and its progression. Oxygen flow was used in a limited number of our patients, namely infants, due to their young age and difficulties in using an appropriate interface for their non-invasive support. CPAP was also used in a small number of patients to manage OSA, but this was in older studies, and current recommendations advise BiPAP use in such patients [4,31], as CPAP does not support ventilation and may further stress respiratory muscles leading to fatigue [3,4,31].

We used objective clinical parameters to assess the presence of SDB. We postulated that the decline in respiratory muscle function and upper airway obstruction in patients with MD which causes SDB could correlate with the decline in pulmonary function test measures and respiratory muscle strength, decreased LVEF, non-ambulation, or the presence of obesity, which is a known risk factor for OSA [4,16,19,32,33,34]. The sensitivity and specificity varied among these measures; the NPV was limited but the PPV was high. This indicates that if any of these measures are present (positive) there is a higher chance for the patient to have SDB, which could help physicians determine the patients who need polysomnography evaluation. However, given the low NPV, their absence is not a good indicator for the absence of SDB, and physicians should make decisions for polysomnography referrals based on the type of MD, progression of disease, age, and SDB symptoms in combination with these predictors.

Muscular dystrophies can lead to significant systemic impacts. As the disease progresses, respiratory muscles become weaker, impairing ventilation and causing SDB, which can eventually lead to respiratory failure [3,14,17]. This is a prominent issue in Duchenne patients, where respiratory failure occurs in later stages of the disease, and in Congenital MDs, where patients can develop respiratory failure in infancy [6,7]. Similarly, cardiac involvement is also a hallmark of MDs, particularly in Duchenne and Becker, where dystrophin deficiency leads to dilated cardiomyopathy [1,7,8]. Given the progressive nature of MDs and the involvement of various organs, early diagnosis and intervention are critical. Current management strategies are largely supportive and include physical therapy, cardiac care, respiratory support, and, in some cases, gene therapies that aim to restore functional proteins or address the underlying genetic defect [1,2,35,36]. Additionally, corticosteroids have been shown to slow muscle deterioration in patients with Duchenne, and recent advances in exon-skipping therapies and gene editing techniques offer hope for more effective treatments in the future [35,36].

Sleep disordered breathing is common in children with MDs primarily due to respiratory muscle weakness and upper airway obstruction, but craniofacial features may also contribute [3,14,37]. Children with Duchenne or Myotonic dystrophy often exhibit altered orofacial development, including features such as steeper mandibular planes, narrower dental arches, and higher palatal vaults, likely secondary to chronic muscle weakness during development [37,38]. These structural differences can reduce upper airway dimensions, increasing the likelihood of obstructive events during sleep. Therefore, part of the risk for SDB and the variability observed among MD patients may reflect the combined effects of muscular and structural factors. Craniofacial changes were not systematically documented in the medical charts and thus could not be analyzed in this study; however, future studies should consider including cephalometric or craniofacial imaging assessments alongside functional respiratory measures to better understand their relative contributions to SDB in MD patients.

The consequences of SDB can be profound; it affects sleep quality with reduced efficiency and frequent arousals, it has deleterious effects on growth, cognitive development, and the cardiovascular system, and its onset could be insidious, with no standardized screening protocols to allow for timely diagnosis [3,4,14,34,39,40]. Thus, it is essential to identify the patients at risk and to evaluate them for SDB by performing an overnight PSG study. Polysomnography can also help determine the optimal management of SDB; a split night or a titration study can help identify the appropriate pressures needed for nocturnal support [3,14,17].

Prior reports documented the high frequency of SDB in MDs while focusing more on Duchenne, which is the most common disease among the group [15,33]. A single-center retrospective study published in 2022 [15], reported OSA in 19.4% and nocturnal hypoventilation in 10.4% of pediatric patients with Duchenne. All of the nocturnal hypoventilation patients were non-ambulant, and FVC < 50% was associated with nocturnal hypoventilation with a sensitivity and specificity of 73% and 86%, but the PPV was limited to 32%. A study by Sawnani H et al. [33] examined pediatric patients with Duchenne being treated with steroids and reported OSA in 63.6%, central apnea in 33.6%, and hypoventilation in 17% of the subjects. They also reported positive correlation between the obstructive apnea index and BMI, and that lower FVC was associated with increased risk of hypoventilation.

Studies focusing on the less common MDs are limited but also reported high prevalence of SDB [14,29,41]. A study focusing on children with type 1 Myotonic dystrophy reported 42% prevalence of sleep apnea and 45% of nocturnal hypoventilation [29]. Another study evaluated polysomnography data for 31 adults with Facioscapulohumeral MD and found 17 patients with OSA (55%) and 8 with nocturnal hypoventilation (26%) [41]. A recently published article analyzed sleep studies for pediatric patients with five different MDs and reported sleep apnea in 73% of the patients. They also found hypoventilation in 43% of the subjects with an observed higher prevalence in the Congenital group (67%) [14].

Our study focused on pediatric patients with MDs, including seven different types, for a relatively large number of subjects. We used standardized definitions for SDB subtypes and objective measures to predict SDB. It highlighted key differences between MD types, and revealed the age at diagnosis, which could be used in combination with the clinical predictors we analyzed to identify patients with or at risk for SDB. This should support medical decisions when making referrals for PSG and allow for early diagnosis and management. Nevertheless, limitations include the retrospective design of this analysis and reliance on single-center data, which may limit generalizability. The subjects included in the analysis were patients referred for PSG evaluation, which probably did not include all the patients with MDs followed at our center. Also, the limited number of patients in certain rare MDs did not allow for further comparisons, and we could not assess clinical predictors for each SDB subtype.

We concluded that OSA is the most common type of SDB among children with MDs. The age at diagnosis and required BiPAP pressures for management vary among the different types. Examining the patients’ PFTs, echocardiography, and ambulatory status may help identify patients with the disease.

Future prospective studies with larger multi-center cohorts should investigate the longitudinal progression of SDB across different MD types to establish clearer guidelines for early detection and intervention and to further address less common diseases.

## Data Availability

The datasets generated and analyzed during the current study are available from the corresponding author, M.A., upon reasonable request.

## Abbreviations

The following abbreviations are used in this manuscript:

ANOVA	Analysis of Variance
BMI	Body Mass Index
CAI	Central Apnea Index
ETCO_2_	End Tidal Carbon Dioxide
FVC	Forced Vital Capacity
ICSD-3	International Classification of Sleep Disorders Third Edition
LVEF	Left Ventricular Ejection Fraction
MD/MDs	Muscular Dystrophy/Muscular Dystrophies
MEP	Maximum Expiratory Pressure
MIP	Maximum Inspiratory Pressure
NPV	Negative Predictive Value
OAHI	Obstructive Apnea Hypopnea Index
OSA	Obstructive Sleep Apnea
PEEP	Positive End Expiratory Pressure
PFT	Pulmonary Function Test
PIP	Positive Inspiratory Pressure
PPV	Positive Predictive Value
PSG	Polysomnography
SDB	Sleep Disordered Breathing
SD	Standard Deviation
T&A	Tonsillo-adenoidectomy
TLC	Total Lung Capacity
TST	Total Sleep Time
